# Parental involvement in zero-separation care: experiences and needs of parents of late preterm infants

**DOI:** 10.3389/fped.2026.1913778

**Published:** 2026-07-29

**Authors:** Kelly Janssens, Bieke Bollen, Annick Bogaerts, Sophie van der Schoor, Sylvia Obermann-Borst, An Eerdekens, Neeltje Crombag

**Affiliations:** 1 Department of Neonatology, University Hospitals Leuven, KU Leuven, Leuven, Belgium; 2 REALIFE Research Group, Women and Child, Department of Development and Regeneration, KU Leuven, Leuven, Belgium; 3 Department of Woman and Baby, University Medical Center Utrecht, Utrecht, Netherlands; 4 Care4Neo, Neonatal Patient and Parent Advocacy Organization, Rotterdam, Netherlands

**Keywords:** attachment, bonding, kangaroo care, late preterm infants, parent–infant closeness, zero separation

## Abstract

**Aim:**

This study explores parents' experiences regarding involvement in the care of their late preterm infants within a zero-separation care model at the semi-intensive care unit (level II) of the University Hospitals Leuven.

**Methods:**

A qualitative design was used, employing in-depth, semi-structured interviews with thirteen parents of late preterm infants recruited between February and September 2025. Data were analyzed using the QUAGOL method and guided by the COREQ framework.

**Results:**

Three overarching themes emerged: Stay at the unit, parental role, and needs regarding involvement in care. Parents described the zero-separation model as highly beneficial for bonding and caregiving, particularly through continuous presence. Over time, routines became familiar and their parental confidence grew. Challenges included inconsistent professional guidance on feeding and unclear communication about medical decisions such as feeding tube placement. Parents appreciated being actively involved in caregiving and valued hands-on coaching. Fathers emphasized their dual supportive role, though some felt overlooked. During prolonged hospital stays, parents experienced intertwined emotional, physical, and social needs, including reassurance, rest, and social connection.

**Conclusion:**

Zero-separation care shows strong potential to enhance bonding, strengthen parental confidence, and foster collaborative family-centered care. Clear and supportive communication, structured guidance, and supportive bedside coaching are essential in meeting parents' evolving needs and to promote parental engagement.

## Highlights

- Zero-separation enabled continuous parent-infant closeness that supported bonding and hands-on caregiving by both parents.
- Inclusive communication, continuity of care, and structured bedside support are essential to optimize parental involvement.

## Introduction

The longstanding practice of separating parents from their preterm born infants immediately after birth has come under increasing scrutiny. In line with this shift, contemporary neonatal guidelines prioritize parent–infant closeness as a foundational developmental objective (1–3). Early, sustained proximity promotes neurobiological maturation, supports autonomic stability, and strengthens early attachment (4–8). Conversely, postnatal separation may contribute to maternal psychological distress, including heightened anxiety and depressive symptoms (9–11). Separation can also disrupt breastfeeding and early bonding, underscoring the need for care models that promote closeness and parental involvement from birth onward (12–15).

The zero-separation model emphasizes continuous physical and emotional closeness between parents and their newborns from the moment of birth. As a fundamental element of the Infant and Family-Centered Developmental Care (IFCDC) framework it has been shown to be both safe and beneficial for infants and parents (2, 16). To clarify the core components and their interrelationships within IFCDC, the Belgian Federal Knowledge Center (KCE) conducted a concept-mapping process, resulting in a flower diagram that summarizes the framework's operational elements (17). Practices such as skin-to-skin contact (kangaroo care) and breastfeeding are essential pillars of IFCDC, providing physiological, immunological, and neurodevelopmental benefits while strengthening parent–infant closeness (18–21).

Despite these benefits, implementation of zero-separation is hindered by structural, organizational, and cultural barriers (22), including limited physical infrastructure (e.g., lack of couplet-care rooms), insufficient interprofessional collaboration and parental uncertainty about their caregiving role (23).

Although the importance of proximity and involvement is well recognized, few qualitative studies have explored how parents of preterm infants experience zero-separation care or what facilitates or hinders it. Evidence is also limited regarding strategies to empower parents to assume caregiving from birth and how teams can enable this. Therefore, this qualitative study explores how parents of late preterm infants experience involvement in care during a hospital stay within a zero-separation policy, identifying their needs alongside the facilitators and barriers to the development of their parental role.

## Methods

We used a qualitative design with in-depth, face-to-face semi-structured interviews to explore parental experiences within a zero-separation model in a semi-intensive care unit. This study was conducted in the Level II unit of the University Hospitals Leuven, which operates as part of the Level III Neonatal Intensive Care Unit (NICU). Immediately following birth in the delivery room, newborns are placed skin-to-skin with the mother. This physical contact is maintained during transport to the neonatal unit. Depending on the situation, the infant is transferred either skin-to-skin with the mother in her bed or accompanied by the father in a wheelchair. The unit where the respondents stayed consists of three couplet care rooms and four Single Family Rooms (SFR's). In the couplet care rooms two separate teams, maternity and neonatology are responsible for providing care with one team attending to the mother and the other to the baby. Once transferred to the SFR, typically when the mother is discharged from the hospital, the neonatology team assumes responsibility for the care of the entire family. In both type of rooms sleeping arrangements (sofa bed) are made possible for the fathers. Care is guided by IFCDC principles, as encouraged by the Belgian government and aligned with international standards (3, 24).

Participants were recruited through convenience sampling and invited face-to-face to participate in the qualitative study. Eligible participants were mothers^1^ aged ≥18 years who had delivered their infant(s) between 34 and 37 weeks' gestation; infants required adequate postnatal adaptation without intensive care. Participants needed to be native Dutch speakers. To gain a broader understanding of family experiences, the fathers were invited to participate in separate interviews, offering complementary perspectives. We excluded pregnancies with fetal congenital anomalies and mothers with known psychological vulnerability.

Ethical approval was obtained from the Ethics Committee of the University Hospitals Leuven (no. S66865) and all participants provided informed consent. Confidentiality and anonymity were assured by assigning pseudonyms and securely storing data on the hard drive of the hospital.

Prior to data collection, the initial version of the interview guide was piloted with a mother who had previously stayed in a couplet care room. This pilot face-to-face interview served to assess the clarity, flow, and relevance of the questions in relation to the study's aims. Data were collected between February and August of 2025. All but the pilot interview were conducted during hospitalization of the baby. Interviews were conducted by a researcher/midwife (KJ) experienced in neonatal nursing. Although the researcher is professionally involved in neonatal care as a NIDCAP trainer, she had no prior clinical relationship with the participants and was not part of the team that cared for the respondents. Interviews were conducted in Dutch. The researcher drew on her clinical expertise and familiarity with the care context to facilitate open and empathetic conversations, while remaining aware of how this background could shape the interaction and participants' responses. While the interview guide provided structure, participants were encouraged to speak freely and elaborate on their experiences, allowing for rich, emergent data. Interviews were conducted separately with mothers and fathers to capture potentially divergent viewpoints. At the start of each interview, participants were informed about the researcher's role and the study aim. The researcher emphasized her interest in the participant's unique experience. She expressed appreciation for their willingness to participate and reminded them that participation was voluntary, that they could decline to answer any question, and that they could stop the interview at any time without consequence. To minimize potential social desirability bias, an open and non-judgmental interviewing approach was adopted. The interviews were scheduled at the participant's convenience and audio recorded. Most interviews took place in a quiet room within the neonatology department, where ambient table lighting created a calm and comfortable atmosphere. This setting helped maintain focus for both the interviewer and participants, while minimizing potential interruptions from staff or the infant. Choosing a separate room also helped preserve the authenticity of the interaction by reducing the potential influence of a father's presence. Two interviews were held in a SFR with the baby present in accordance with the mothers' wishes. Although the baby's presence could have posed a potential distraction, it did not interfere with the interview process. During one of those interviews, the father was present for the first few minutes. The pilot interview was conducted at the respondent's home, in the living room, also with the baby present, 6 months after discharge. Interviews were audio recorded and transcribed verbatim by a professional service (Amberscript Global BV, Amsterdam, the Netherlands). Median duration was 25 min (range 17–42 min).

The interview guide supported in-depth exploration of parents' experiences and perceptions of their involvement in neonatal care. The guide is detailed in Supplementary Appendix S1.

The guide was reviewed by an experienced qualitative researcher (NC) to ensure methodological rigor and alignment with the study's objectives. This researcher also participated in the data triangulation process, critically reviewing emerging themes and interpretations. Collaborative discussions helped refine thematic boundaries and validate interpretations, thereby strengthening the credibility and depth of the findings. In addition, the interviewer kept reflective field notes before and after each session, documenting impressions, emotional responses, and non-verbal cues, in line with best practices for qualitative research rigor. Data interpretation and analysis followed Quagol (Quality Assessment Guide for Qualitative Studies) (25). The whole trajectory of data interpretation and concurrent analysis was discussed with an experienced researcher (NC) in the field of qualitative studies. In a first phase, transcripts were read repeatedly to achieve an in-depth understanding of each participant's narrative. For each transcript, various reports were subsequently prepared, including a descriptive, methodological, and content report, to aid in data analysis and enhance the yield of subsequent interviews. The findings were consistently and transparently reported. Key concepts were identified to capture the underlying meaning of the care context for parents. In addition to defining the key concepts that come at play, future recommendations for advancing the care for parents and their preterm born babies guided this research. The process was continued until data saturation was reached; no new information was obtained from subsequent interviews and thus recruitment was stopped (26). NVIVO (v15.1.2, Lumivero, Denver, CO, USA) was used for data organization and coding. The COREQ (Consolidated Criteria for Reporting Qualitative Research) framework guided study reporting.

## Results

Thirteen parents participated (*n* = 7 mothers; *n* = 6 fathers). The demographic characteristics are detailed in Supplementary Appendix S2. All interviews, except the pilot interview, were conducted during hospital admission. The pilot interview was not originally intended for inclusion. However, it provided important insights, particularly on breastfeeding. These findings were consistent with those from the interviews conducted during hospital admission. The pilot interview was therefore included in the analysis, specifically to inform the breastfeeding theme. Thematic analysis identified three overarching themes: stay at the unit, parental role, and needs, comprising ten subthemes. Subthemes further elaborated how parents experienced involvement in their infant's care. An overview can be found in Tables 1–3. Themes are presented as headings. Respondent quotes are identified with F (father) and M (mother).

**Table 1:** Subthemes and illustrative codes regarding stay in the unit/rooming-in.

Theme	Subthemes	Illustrative codes
STAY IN THE UNIT/ROOMING-IN	Evolving over time	Initial overwhelm Adaptation over time
	Uncertainty	Lack of clarity around medical decisions Anticipated length of stay Ambiguity feeding progress
	Environment	Disturbing elements ○ Frequent hospital care professionals (hcp) visits in beginning, at night ○ (Unnecessary) noise from medical equipment Facilitating elements ○ Benefits of the non-separation context (being able to stay together, sleeping arrangements, availability fridge,.) ○ Privacy

**Table 2:** Subthemes and illustrative codes regarding parental role.

Theme	Subthemes	Illustrative codes
PARENTAL ROLE	Involvement	Active participation in care Meaningful division of roles between parents ○ Father takes on supportive and caring role—“dual role fathers” There is a shared sense of responsibility and presence Building autonomy
	Breastfeeding mothers	Concerns/uncertainty Support for breastfeeding and pumping is crucial ○ Need for guidance ○ Reliance on specific staff members Communication about feeding tube and implications Fatigue
	Bonding and proximity	Through skin-to-skin contact starting birth Continuity of presence as key to bonding ○ Deep satisfaction from being constantly present with the baby Desire for bonding ○ Need for joint early bonding ○ Fathers emphasize the importance of being present to build a bond, often taking parental leave to do so Through feeding and other caregiving tasks

**Table 3:** Subthemes and illustrative codes of needs regarding involvement in care.

Theme	Subthemes	Illustrative codes
NEEDS REGARDING INVOLVEMENT IN CARE	Support	Practical support ○ Direct advice in caregiving—education ○ Learning to interpret baby's signals ○ Appropriate autonomy ○ Recognition of both parents as caregivers Mental/psychosocial support ○ Emotional support during moments of exhaustion and pain ○ Need for feedback—reassurance ○ Feeling isolated when admitted longer period ○ Need for “air”
	Continuity of care	Desire for fewer, more consistent caregivers Need for consistent messaging and follow-up across caregivers Need for early orientation on team structure and responsibilities
	Communication	Around medical decisions Also directed at the father
	Shared decision-making	Lack of early involvement in decision-making

### Stay at the unit/rooming-in

Stay at the unit refers to the continuous presence of parents near their late preterm infant in a setting that enables rooming-in without separation, allowing physical and emotional presence and active participation in care while bonding.

#### Evolving over time

Parents described the first days as intense: balancing physical recovery, breastfeeding and pumping, learning new care tasks, and frequent staff entries. Some fathers hesitated to leave the room out of fear of missing information or care moments.

“That first night was really tough. We had only slept four hours the night before because of all the tension. […] We had to start feeding, then manually pump, times two. I think we were busy for almost two hours, and three hours later they were back again. I had a splitting headache. I think I slept maybe two hours that whole night. […] I didn't even dare to leave the room.” (R4, F)

Over time, routines became familiar and confidence grew.

“And then it's about getting in sync with each other, right? Maybe already creating some routines. Going home will still be a challenge, and we'll have to find new routines again. But maybe we've already established some basic ones here.” (R2, F)

#### Uncertainty

Uncertainty centered on the anticipated length of stay, clarity around medical decisions (e.g., feeding-tube placement), expectations regarding caregiving responsibilities, and ambiguity about the feeding progress. Parents struggled with the absence of even an approximate timeframe for discharge, which complicated logistical planning and emotional adjustment. Some felt unclear about, or insufficiently involved in, decisions such as tube placement. Parents were also sometimes unsure when they were expected to take over specific tasks and who was responsible for what.

“We started to participate, but we didn't really know exactly when we were expected to take over everything ourselves. Sometimes we found it difficult, like, ’Should we already be doing this on our own?’ […] There were things we just didn't know, which made us worry that maybe we had done something wrong.” (R12, M)

Feeding was a major source of uncertainty—both regarding how much the baby was fed, and whether progress was adequate—and it caused anxiety, especially in the context of discharge preparation.

“On the first day, they quite quickly started talking about a feeding tube. […] And that it wasn't explained that the tube was needed to help build up breastfeeding, and that they could have clarified, like, ‘okay, this is the tube, but the goal is still breastfeeding.’ Yeah, that was a bit of a shock.” (R3, M)

#### Environment

The physical and sensory environment strongly shaped parents' experiences. Parents valued the possibility to stay close, with privacy, 24/7 presence and appropriate partner sleep arrangements. Room facilities such as a private bathroom and a small fridge were appreciated. Yet parents often found it challenging to achieve sufficient rest and avoid sensory overload.

“With a bit of imagination, you could say it felt like being on holiday in a little apartment, and that you, that you just, uh, didn't have to do anything that day, and yeah, you just took care of each other.” (R2, F)

“In the beginning, those alarms were going off constantly because they would drop quickly, there was nothing wrong, but their heart rate would dip fast, even though their oxygen levels stayed fine. But yeah… especially at first, and you're not used to those alarms, you're hyper-alert. So I'm telling you, it was really… pffff… super stressful in the beginning (laughs).” (R4, F)

### Parental role

Parental role refers to how mothers and fathers perceive, develop, and enact their caregiving identities within zero-separation care. Three interrelated subthemes emerged: involvement, breastfeeding, and bonding & proximity.

#### Involvement in care

Parents wanted to be actively involved in hands-on care—feeding, changing, bathing, comforting—as both a practical contribution and as a key marker of their parental identity. Even when maternal recovery (e.g., after caesarean) delayed full participation, their eagerness to assume caregiving tasks was strong.

“The boys absolutely didn't like their first baths. And then they told us to try using a swaddle cloth, so they would feel more snug in the bath. Yes, and then we tried that, again today, and you can see them enjoying the bath more and more. So I think that's really nice. And also that, um, they first explained it to us really well, and now they're giving us the chance to do it all ourselves. Because we'll have to do it on our own at home too, right?” (R5, M)

Couples developed shared rhythms and adapted roles over time, fostering a sense of teamwork and mutual support. Fathers described a dual role—providing active infant care while also supporting their partner—a pattern that was especially pronounced after caesarean birth.

“For me as a father, it's not just about caring for the babies, but really everything around it as well. Uh yeah, also making sure my wife can focus on the care. Uhm, and that I take on the supporting role as much as possible […] and of course, since it's twins, I also help with the care. Uhm yeah, it's really a bit of both, right? Making sure my wife can focus as much as possible on caring for them, and that she doesn't have to worry too much about other things.” (R2, F)

Over time, staff encouragement and opportunities to take over tasks independently supported parents' sense of autonomy.

“In the first few days they really take it out of your hands, but then they gradually let go. And now they ask me what I want to do.” (R3, M)

#### Breastfeeding (mothers)

Breastfeeding was central yet challenging; particularly regarding milk supply, positioning, and transitioning from tube to breast. Fathers supported mothers by assisting with pumping and providing emotional encouragement. Staff offered reassurance and practical tips, although support was sometimes inconsistent, particularly for exclusive breastfeeding, prompting reliance on specific staff members. Clear communication about feeding strategies (breast, bottle, tube) increased parental confidence. Despite exhaustion from pumping and night-time routines, motivation to continue breastfeeding remained high.

“Yeah, I was really exhausted. Because at night they told me, for example, ‘try to sleep and we'll give tube feeding,’ and I wanted to, but my breasts couldn't handle that, so I had to pump at night anyway. So then I also felt it was a shame if he got tube feeding […] I let him have it, but he would often wake up during the feeding, so I preferred if he was held upright or something. So then I took him with me, again (laughs). […] Then I pumped after the feeding had finished, and that meant I was busy for a long time again… Yeah, I found that really exhausting.” (R1, M)

#### Bonding and proximity

Continuous presence facilitated parent-infant bonding and responsive caregiving. Parents valued immediate and ongoing skin-to-skin contact. Some fathers wished for more structured opportunities. Routine caregiving was experienced as meaningful and confidence-building.

“Yes, it definitely helps to always be together. You start to get to know them gradually. Now they're starting to respond more, and when they're in their little beds, it's a different environment for them. They make new sounds, have restless moments… and being close really helps to get to know them” (R3, M)

One father described being alone with his infants shortly after birth, while his partner remained in the delivery ward for an hour; an unsettling separation during a critical bonding window that highlighted the value of joint early bonding as a caregiving couple, provided there is clear explanation and communication.

“After the birth, she still needed to regain her strength […] I was taken to the room with the babies on my chest, in a bed, well, a chair, and they left me there alone for an hour. Probably because my wife still… well, had to recover emotionally, […]. Yes, but she felt bad about that, and I also found it really upsetting. That moment lasted quite a while, because I didn't know these little beings. I didn't know what fatherhood meant to me… There were nurses, […], but still, it took another hour before my wife was there… And I find that so hard… Why can't we just stay together?” (R6, F)

### Needs regarding involvement in care

Parental needs—emotional, informational, and practical—were central to how parents made sense of zero-separation, especially regarding support, continuity of care, communication and shared-decision-making.

#### Support

Parents needed practical, hands-on support, especially from admission onwards. Demonstrations followed by supervised practice built confidence and reduced anxiety. Understanding the rationale behind recommendations was important, as did jointly observing and interpreting infant cues with staff. As confidence grew, parents desired increasing autonomy with staff remaining available. One father recounted being reprimanded for initiating gavage feeding, underscoring the need for clearer recognition of parents' caregiving role. Parents wanted to be acknowledged as equal caregivers; fathers in particular valued being actively included in skin-to-skin contact, feeding, and comforting.

“Uhm, but what's really great about that unit is that you can do as much of the care yourself as you want, sometimes even a bit more than what's technically allowed. For example, if the bottle wasn't finished and there were still 10–15 milliliters left, I once just put it into a syringe and gave it myself. And then the nurse said, ‘You should really leave that to us.’ But I thought, well, what's the risk? […] For other things you can do yourself, it also means less staff are needed for every task, because some responsibilities are shared with the parents, not forced, but if they want to do it… Uhm, then it's possible, and the staff can be available to help in other rooms, you know?” (R2, F)

The emotional intensity created a need for psychosocial support and reassurance. Parents benefited from empathetic caregivers, occasional respite during long stays, and opportunities for social contact; while mothers staying alone sometimes felt isolated.

“Sometimes you just want to see someone and talk. Especially when your stay gets longer, it would help if someone would proactively offer a chat with a psychologist, even just once, because it's hard to ask for that yourself as a new mother. You quickly feel like you're failing […] It's really hard to be alone all day. After my partner left, I felt like I fell into a black hole. I even called my mother late at night to come stay with me, because after several days together, being alone was just too much. I think I'm slowly getting used to it, but it's not easy.” (R11, M)

#### Continuity of care

Staff changes sometimes led to inconsistent messaging and disrupted trust and familiarity. Conflicting advice—particularly about feeding—was confusing. Stable teams who knew families' stories and preferences were experienced as reassuring. Parents suggested more structured handovers that actively included them, as well as early orientation to team roles.

“…if the midwife who was with me the whole shift could just come and say, ‘My shift is over, let me quickly go over things, these were the most important points today, I've handed over this and that.’ And then ask, ‘Are there any other things you're thinking about?’ […] so that we could maybe take a moment to wrap things up together, and you'd know, soon a new face will come in and we're on the same page… I think that would have given me a lot of peace of mind.” (R9, M)

#### Communication

Parents wanted clear, timely explanations of what was happening and why, including changes in their infant's condition, routines, or procedures. Fathers noted that they were not always addressed during updates and emphasized inclusion in the communication.

“Just what I said a moment ago, that I do indeed understand. From a certain point onwards, they look much more to the mother for breastfeeding and so on. Where you yourself are then… It's not that this is the case for everyone, but with some, you get the feeling that they, Uhu…. Burden is a very strong word to use, but that they think that the father isn't really that important to be there or something … with others, you get a completely different feeling … To give a simple little example, when someone comes in, when you've pressed the bell or whatever, they immediately look at the mother, like, ‘Yes, what do you need?’, when it could actually have been me…” (R13, F)

#### Shared-decision making

Parents wanted meaningful involvement in decisions, rather than receiving information only after choices had effectively been made—especially regarding tube feeding and discharge timing.

“But by the time something is presented to you, it's almost not a choice anymore… it's not like, ‘Okay, now we're going to discuss how you feel about it.’ That really caused me quite a bit of stress.” (R10, F)

## Discussion

This study provides insights into how parents of late preterm infants experience care within a zero-separation model. Continuous presence and couplet care supported bonding, intuitive caregiving, and parental empowerment. Single-family rooms, private amenities, and uninterrupted proximity fostered emotional closeness and responsive caregiving, in line with an oxytocin-based paradigm that links continuous parent-infant contact to neurobiological regulation and bonding (27).

Challenges were most prominent early in the stay when the combined burden of physical recovery, information overload and unclear communication undermined parents' confidence. Continuity of care mattered greatly: mothers valued a stable team familiar with their story. Having a known healthcare provider is central to relational continuity, which enhances trust, promotes familiarity, and facilitates more effective communication (28). These insights are particularly pertinent in the couplet care context, where multiple professionals are involved in both maternal and neonatal care (29). They underscore the importance of structured handovers and transparent communication strategies such as bedside briefings to ensure that parents feel informed and supported.

Although parents described skin-to-skin contact as a meaningful way to care for their infant and to foster emotional bonding, duration often fell short of WHO recommendations (2), as parents described having skin-to-skin contact immediately after birth and during the early days of hospitalization. This appeared partly due to limited structured support and encouragement despite physical proximity.

Parents accounts of inconsistent breastfeeding support echo earlier work that proximity alone is not sufficient. Breastfeeding, a central yet demanding experience, requires consistent, cue-based lactation support and clear communication, particularly for exclusive breastfeeding and for late preterm infants who often struggle with feeding (30, 31). Tube feeding should be framed positively in communication with parents as a temporary, supportive step, ideally addressed prenatally and reinforced with visual aids in the room to reduce anxiety and support developmental care.

Fathers in particular, described a dual role—active infant care alongside support for their partner-yet sometimes felt overlooked in communication and in opportunities for skin-to-skin contact and feeding. This aligns with growing evidence that fathers seek active involvement in neonatal care and benefit from inclusive practices that explicitly address gendered expectations (32). Our results suggest that deliberate inclusion of fathers in everyday care, communication, and decision making is a key lever to fully realize the potential of IFCDC (33).

Peer support and a chosen support person may reduce isolation during longer stays (34). Parent-infant proximity, while essential is not sufficient on its own. Empowering families within zero-separation neonatal care requires full implementation of IFCDC—inclusive communication, continuity of care, structured and psychosocial support—to optimize outcomes.

Limitations of this study include a small sample size, which may limit transferability of the findings. Nearly half of the participants were healthcare professionals, which may have deepened reflections on communication and clinical routines but also introduces bias in perceptions of care and communication. Due to these limitations, the findings strongly reflect a local culture that actively facilitates parental presence, and their generalizability to neonatal settings without similar structures may be limited, as such contexts may face different barriers. One pilot interview was included in the analysis. Although conducted outside the planned study procedures, its findings were consistent with the main dataset and contributed to the breastfeeding theme. Most participants were highly educated and, except for one mother, white Caucasian, reducing diversity and transferability to broader socio-economic and cultural contexts. Caring for twins was relatively common which may also have intensified the experiences, particularly regarding fatigue and sensory overload. Strengths of the study include its focus on a specific population within a fully operational zero separation care model, the inclusion of both mothers and fathers and the qualitative study design.

## Conclusion

This study explored how parents of late preterms experience care and involvement in a zero-separation care model. Continuous presence and couplet care supported early bonding and its role in empowering parents during the transition to parenthood. However, identified gaps in care highlighted the need for structured support to strengthen family partnerships. At the bedside, clear anticipatory communication and shared decision-making emerged as key components of this support. Staff training should focus on inclusive relational care that actively recognizes both mothers and fathers as equal caregivers and supports them in taking up their roles. Accessible psychosocial support should be proactively integrated, especially for parents during prolonged hospitalization. Together, the themes illustrate how specific environmental, relational, and organizational factors can either facilitate or hinder parents in growing into their caregiving role under the umbrella of zero separation care.

## Data Availability

The datasets presented in this article are not readily available to preserve participant anonymity, as the data contains potentially identifiable qualitative information, the datasets are not publicly available. Data are available from the corresponding author on reasonable request. Requests to access the datasets should be directed to kelly.janssens@uzleuven.be.

## Footnotes

^1^For the sake of fluency and readability the term ‘mother’ will be used throughout this manuscript to refer to the birthing parent regardless of gender identity; The term ‘father’ is used to refer to the other parent, as all individuals in this role identified as male.
