# Supplementary material for: Parental involvement in zero-separation care: experiences and needs of parents of late preterm infants
Source: Front Pediatr. 2026 Jul 29;14:1913778. doi: 10.3389/fped.2026.1913778 (PMC13462383; doi:10.3389/fped.2026.1913778)
Supplement: Supplementary file 1 [file Supplementaryfile1.docx]

# Interview guide

## Introduction

### Introduction of the interviewer

The interviewer presents herself and makes sure the participant is set right at ease. The participant will be informed about the study and the purpose of the interview. In this part the interviewer will emphasize why she is interested in the unique experience of the participant. She will express her gratitude to the parent for accepting to participate in the study and reminds him or her that it is possible to choose not to answer a question or stop altogether with the interview at any given time. All the information that will be gathered will be pseudonymously stored and will not be traceable back to the participant.

### The aim of the interview

The aim of the interview is to explore how parents experience care of their late preterm infants in a zero-separation care model. What are facilitators and barriers for an optimal involvement in care according to parents? Parents will be asked to share how they take care of their baby in the unit and how they feel supported to do so.

In 2022 the Federal Knowledge Center (FKC) conducted a study on IFCDC in Belgium (Detollenaere et al., 2022). The researchers performed a concept mapping and subsequently developed a flower diagram of the main elements to deliver IFCDC. The visualization as a flower highlights the importance that all concepts stem from a general center; the preterm infant and his family. The main elements are interconnected “mutually reinforcing and blending into what is defined as a philosophical approach and set of principles to guide the delivery of neonatal healthcare”. An overview of the elements is presented in Figure 1 (Detollenaere, et al., 2022).

The themes that will emerge inductively from the interview data will be matched with the core elements specified in the flower diagram developed by the FKC. The facilitators and barriers will be identified accordingly.

**Figure 1**

*Flower diagram with elements to deliver IFCDC*

Note. From “Infant- and family-centred developmental care for preterm newborns in neonatal care” by J. Detollenaere et al. 2022, Health Services Research (HSR) Brussels: Belgian Health Care Knowledge Centre (KCE). KCE Reports 350, p.18 (<https://kce.fgov.be/sites/default/files/2022-03/KCE_350C_Mother-child-care_Synthese.pdf>). Reprinted with permission.

## Questions

### Opening questions

- What does being involved in your baby’s care mean to you personally? / Wat betekent

betrokken zijn bij de zorg van je baby voor jou persoonlijk?

- In what ways do you take care or do you participate in the care of your baby? / Op welke manier zorg je of neem je deel aan zorg voor je baby?

### Key question

- How did you experience this (care) on our unit? Hoe heb je dit (deze zorg) ervaren op onze eenheid?

During the interview when aspects of the flower diagram such as,

- architecture
- parental support, guidance and education
- parental presence and caregiving
- health care staff, time and education
- technology based support
- shared decision making
- or transition periods

come up spontaneously, the interviewer will try to zoom in on them accordingly. Facilitators and barriers will be questioned.

For example if the participant talks about breastfeeding, next to enquiring to the parents’ experience, the intention to breastfeeding can be questioned, the enabling elements and not helping elements to achieve breastfeeding in our unit.

### Follow-up questions

- Can you elaborate? Kan je daar meer over vertellen?
- Can you give other examples? Kan je andere voorbeelden geven?

### Closing questions

Do you have any advice for other parents who stay with their baby in this unit?/ Heb je advies voor andere ouders die met hun baby in deze eenheid verblijven?

Are there any issues or concerns that are important to you that we have not discussed?/Zijn er andere belangrijke zaken of bezorgheden die we niet besproken hebben?
