# Supplementary material for: Parental involvement in zero-separation care: experiences and needs of parents of late preterm infants
Source: Front Pediatr. 2026 Jul 29;14:1913778. doi: 10.3389/fped.2026.1913778 (PMC13462383; doi:10.3389/fped.2026.1913778)
Supplement: Supplementary file 2 [file Supplementaryfile2.docx]

| **Resp ID** | **Mother / Father** | **Partner participated** | **Interview day after birth** | **Discharge day after birth** | **Gestational age wk** | **Delivery mode** | **Twins** | **Breastfeeding** | **Occupation** | **Age** | **Child number** | **Previous premature births** |
| --- | --- | --- | --- | --- | --- | --- | --- | --- | --- | --- | --- | --- |
| 1 | Mother | No | > 19 (pilote) | 19 | 34 3/7 | Primary caesarean | No | Exclusive | Employee | 29 | First child | No |
| 2 | Father | Yes | 11 | 17 | 35 1/7 | Primary caesarean | Yes | Mixed | Employee | 32 | First children | No |
| 3 | Mother | Yes | 11 | 17 | 35 1/7 | Primary caesarean | Yes | Mixed | Employee | 35 | First children | No |
| 4 | Father | Yes | 12 | 23 | 36 1/7 | Primary caesarean | Yes | Mixed | Independent | 31 | First children | No |
| 5 | Mother | Yes | 12 | 23 | 36 1/7 | Primary caesarean | Yes | Mixed | Employee | 29 | First children | No |
| 6 | Father | No | 10 | 19 | 35 3/7 | Vaginal birth | Yes | Mixed | Employee | 32 | First children | No |
| 7 | Mother | Yes | 7 | 7 | 36 6/7 | Secondary caesarean | No | Mixed | Employee | 32 | Second child | Yes, second time in couplet care |
| 8 | Father | Yes | 7 | 7 | 36 6/7 | Secondary caesarean | No | Mixed | Employee | 34 | Second child | Yes, second time in couplet care |
| 9 | Mother | Yes | 7 | 15 | 36 6/7 | Vaginal birth | No | Exclusive | Employee | 31 | First child | No |
| 10 | Father | Yes | 8 | 15 | 36 6/7 | Vaginal birth | No | Exclusive | Employee | 31 | First child | No |
| 11 | Mother | No | 10 | 23 | 35 4/7 | Vaginal birth | No | Mixed | Employee | 33 | First child | No |
| 12 | Mother | Yes | 21 | 22 | 34 5/7 | Vaginal birth | Yes | Exclusive | Employee | 29 | First children | No |
| 13 | Father | Yes | 21 | 22 | 34 5/7 | Vaginal birth | Yes | Exclusive | Employee | 30 | First children | No |
